# A much valued tool that also brings ethical dilemmas - a qualitative study of Norwegian midwives’ experiences and views on the role of obstetric ultrasound

**DOI:** 10.1186/s12884-019-2178-x

**Published:** 2019-01-16

**Authors:** Annika Åhman, Kristina Edvardsson, Tove Anita Fagerli, Elisabeth Darj, Sophia Holmlund, Rhonda Small, Ingrid Mogren

**Affiliations:** 1 Department of Clinical Sciences, Obstetrics and Gynecology, Umeå University, 901 87 Umeå, Sweden; 2 Judith Lumley Centre, La Trobe University, Melbourne, Victoria Australia; 3 National Center for Fetal Medicine, St Olavs Hospital, Trondheim University Hospital, Trondheim, Norway; 4 Department of Obstetrics and Gynecology, St Olavs Hospital, Trondheim, Norway; 5 Department of Public Health and Nursing, NTNU, Norwegian University of Science and Technology, Trondheim, Norway; 6 Department of Women’s and Children’s Health, Uppsala University, Uppsala, Sweden; 7 Reproductive Health, Women’s and Children’s Health, Karolinska Institute, Stockholm, Sweden

**Keywords:** Pregnancy, Ultrasonography, Midwives, Qualitative research, Norway

## Abstract

**Background:**

Midwives are the main providers of routine antenatal care services including the routine ultrasound examination in Norway. The ultrasound examination can be perceived by expectant parents not only as a medical examination but also as a social event facilitating attachment to their fetus. This study explores Norwegian midwives’ experiences and views on the role of ultrasound in clinical management of pregnancy.

**Methods:**

A qualitative study design was applied. Twenty-four midwives who all performed obstetric ultrasound examinations were recruited for focus group discussions and individual interviews. Data collection took place in 2015 in five hospitals in two different regions of Norway. Data were analyzed using qualitative content analysis.

**Results:**

Midwives described obstetric ultrasound examinations as very valuable although doing ultrasounds placed high demands on their operational and counselling skills. Increasing requests for ultrasound from pregnant women were mentioned. Advancements in ultrasound diagnosis were considered to have put the fetus in the position of a patient, and that pregnant women declining ultrasound could be viewed as irresponsible by some health professionals. Ethical concerns were raised regarding the possibility of pregnancy termination when fetal anomalies were detected. Fears were also expressed that prenatal diagnoses including those following ultrasound, might create a society where only ‘perfect’ children are valued. However, participants stressed that their intention in performing ultrasound was to optimize pregnancy outcome and thereby assist expectant couples and their unborn children.

**Conclusions:**

Midwives in Norwegian maternity care services describe obstetric ultrasound as very valuable, playing a central role in pregnancy management by optimizing pregnancy outcomes. Although high demands are placed on operators’ technical skills and counseling, midwives described performing obstetric ultrasound as very satisfying work. However, midwives believed that expectant parents’ approach to the ultrasound examination, both its medical value and the precious images obtained of the fetus, could put extra strain on the midwives performing ultrasounds. The potential of ultrasound to detect fetal anomalies and the possibility that this may lead to termination of pregnancy, seemed to create some ambivalent feelings in midwives towards its use.

**Electronic supplementary material:**

The online version of this article (10.1186/s12884-019-2178-x) contains supplementary material, which is available to authorized users.

## Background

Ultrasound has been utilized in obstetric care for several decades and it is now considered a routine pregnancy examination in many countries across the world. The ultrasound examination plays an important role in pregnancy management for estimation of gestational age, amniotic fluid amount, localization of the placenta and early detection of multiple pregnancy [1]. As the technique has developed it is now possible to diagnose a range of different fetal malformations and other adverse conditions [2]. Use of Doppler ultrasound for assessment of umbilical blood flow has also been shown to have the potential to detect placental insufficiency, fetal anemia, and to reduce perinatal death without increasing medical interventions [3]. Further, continued advances in obstetric ultrasound have now made prenatal management of some birth defects or fetal malformations a reality [4]. The fetus can potentially be seen as a patient which may cause ethical dilemmas for the clinician, when on occasion, the pregnant woman’s health interests conflict with those of the fetus [5]. With the advancements in the quality of fetal ultrasound imaging there has been increasing demand also for non-medical ultrasound examinations [6]. In addition, expectant parents may view the ultrasound examination as a social event for establishing contact with their “unborn child” [7]. A cautious approach to ultrasound has been described as necessary however, to ensure safety in fetal development, especially in the use of Doppler ultrasound in pregnancy [2].

The ultrasound examination came into use in Norwegian obstetric care in the 1970s, and it was first introduced as a routine examination in two maternity clinics in 1986 [8]. A National Centre for Fetal Medicine was launched in Trondheim in 1990. In 1996, the centre became a WHO Teaching and Training Centre in Ultrasound in Obstetrics and Gynecology [9]. University courses in obstetric ultrasound including both theoretical and practical training are now available at the centre for both physicians and midwives [10].

In accordance with Norwegian National Health Care Regulations, all pregnant women are to be offered an ultrasound examination between 17 and 19 weeks of pregnancy [11, 12]. Midwives with special training in ultrasound are the main providers of routine ultrasound examinations in Norway, while both midwives and obstetricians perform Combined Ultrasound and Biochemical Screening (CUB). When a fetal deviation is detected, an obstetrician will perform a second opinion ultrasound examination and assess whether there is any need for invasive prenatal diagnosis [13]. The Norwegian regulations also stipulate that first trimester ultrasound and invasive prenatal diagnosis, such as chorion villus sampling (CVS) and amniocentesis (AC) should only be offered to women with conditions related to elevated risk of fetal anomalies, such as being 38 years or more [14]. It is recognized however that the Norwegian regulations have not been consistently followed and that there are private practitioners who view anxiety in women as an indication for performing additional ultrasound examinations [15].

Our previous qualitative research within the CROss Country Ultrasound Study (CROCUS) in Norway has demonstrated that Norwegian obstetricians experience challenges associated with provision of ultrasound and prenatal diagnosis services, including counselling dilemmas and perceived differences in expectations between caregivers and expectant parents [16]. From our quantitative CROCUS Study in Norway we have reported midwives’ and obstetricians’ views on how many ultrasound examinations should be part of standard care during pregnancy [17]. Fifty-eight per cent reported satisfaction with the offer of one scheduled ultrasound examination during pregnancy, as recommended by Norwegian guidelines. Health care professionals who used ultrasound themselves were significantly more likely to want to offer more ultrasound examinations [17].

Although physicians and obstetricians have the overall responsibility for fetal diagnosis, midwives are the main providers of routine antenatal care services and routine fetal ultrasound screening in Norway. In light of these issues and the lack of previous research, it is of interest to explore the experiences of Norwegian midwives performing obstetric ultrasound.

## Methods

### Aim

The purpose of this study was to explore Norwegian midwives’ experiences and views on the role of obstetric ultrasound in clinical management of pregnancy, and in situations where maternal and fetal health interests conflict.

### Study design

The study is part of the international CROss Country Ultrasound Study (CROCUS) [18, 19]. A qualitative study design was applied in the absence of previous studies on this issue and to inform subsequent quantitative work. To understand Norwegian midwives’ experiences and views of performing ultrasound examinations data were collected through five focus group discussions (FGDs) and two individual interviews with midwives performing pregnancy ultrasound in maternity care (*N* = 24). An interview guide designed for the CROCUS study was utilized for both FGDs and interviews. The guide included a set of key domains, including topics on midwives’ perceptions about the value of obstetric ultrasound in clinical management, situations when the fetus may be regarded as a patient, and when maternal and fetal interests may conflict, as well as other ethical aspects (See Additional file 1). Participants were also encouraged to raise additional topics or areas of specific interest related to the aim of the study.

### Participant recruitment

Participants were purposively recruited from five different hospitals in the western and middle regions of Norway to enable a broad and varied range of views from midwives. Two of the included hospitals were university hospitals that were approved nationally to perform ultrasound examinations as part of prenatal diagnosis, and three were regional hospitals. The number of deliveries at the hospitals varied between 500 and 5100 annually. Eligible participants were midwives with varying lengths of experience of performing obstetric ultrasound examinations. The recruitment of participants was assisted by a coordinating midwife at the Fetal Medicine Centre in Trondheim (third author, TF), who contacted the heads of the five selected hospitals by telephone to obtain approval for recruiting participants for the study. Participant information and consent forms were thereafter sent to the included clinics.

### Data collection and analyses

Data were collected in December 2015 through five focus group discussions (FGDs) and two additional individual interviews that replaced a FGD cancelled due to a staff shortage on that particular day (*N* = 24). The data collection was performed at the participants’ work place. Before the start of each FGD and individual interview, the participants were given a brief questionnaire to fill in their background characteristics. Two female members of the research group with no prior relationship with the study participants moderated the FGDs: AÅ, a midwife with a PhD, experienced in undertaking qualitative research interviews, and SH, midwife and PhD student. The interviews, lasted between 59 min and 74 min, and SH performed the two individual interviews lasting 45 min and 19 min, respectively. Field notes were taken to capture contextual information during the data collection. Data saturation, i.e. where no new data emerged, was considered to be achieved after the fifth FGD at the fourth hospital. The FGDs and the interviews were digitally recorded and transcribed verbatim in Norwegian, and data were analyzed using qualitative content analysis described by Graneheim & Lundman 2004 [20]. The interview transcripts were read as a whole, and then those parts comprising information relevant to the study questions were carefully examined by AÅ, enabling meaning units to be identified. These were then condensed and coded manually, and sorted into sub-categories, then grouped to form three main categories. The findings were discussed between the authors until consensus was reached on the interpretation of data, the designation of categories and the presentation of results. Quotations selected for inclusion in the manuscript where translated to English. The COREQ checklist guided reporting of this study (See Additional file 2).

## Results

A total of 24 midwives participated in this study (Table 1). The participants were between 34 and 65 years of age (mean age 52.3 years) and all were females. Their length of work experience in obstetric care ranged between 3 and 37 years (mean 22.6 years). All participants had formal ultrasound training and conducted obstetric ultrasound examinations regularly as a part of their general midwifery practice.

**Table 1:** Characteristics of focus group participants (*N* = 24)

No.	Workplace	Number of participants (n)	Age, mean (range years)	Length of work experience in obstetrics Mean (range years)
1	University hospital	4	52.0 (50–55)	20.8 (15–25)
2	University hospital	3	52.0 (47–60)	23.7 (15–37)
3	Regional hospital	3	61.4 (60–63)	33.7 (30–37)
4	University hospital	7	50.4 (40–58)	19 (3–31)
5	Regional hospital	5	51.0 (34–65)	25.6 (10–36)
6^a^	Regional hospital	2	47.0 (41–53)	12.5 (5–20)
All		24	52.3 (34–65)	22.6 (3–37)

^a^Two individual interviews

The results from the analysis of the FGDs and the individual interviews are described in three main categories I: Ultrasound plays a central role in pregnancy management; II: Ultrasound contributes to the fetus being viewed as a patient; and III: Midwives may have ambivalent feelings about its use. Each category comprised two to three related sub-categories (Table 2). Citations from the FGDs are labelled as FGD 1–5, and citations from the individual interviews are labelled as Interview 6 and 7.

**Table 2:** Categories and their sub-categories

Categories	Sub-categories
I: Ultrasound plays a central role in pregnancy management	• A much valued tool by midwives and pregnant women • Places demands on midwives’ technical and counseling skills
II. Ultrasound contributes to the fetus being viewed as a ‘child’	• The ultrasound creates an image of a ‘child’ • It makes you treat the fetus as a patient • Only ‘irresponsible’ mums decline ultrasound examinations
III. Ambivalent feelings about obstetric ultrasound	• Ultrasound may be perceived as a selection method • Ultrasound examinations are intended to optimize pregnancy outcomes

### Ultrasound plays a central role in pregnancy management

This category describes the participants’ views of the value of ultrasound for midwives themselves and their perception of pregnant women’s views, and also the demands placed on midwives performing obstetric ultrasound. This category is described in the following two subcategories.

#### A much valued tool by midwives and pregnant women

The participants described ultrasound as having a central role in medical management of pregnancy. Ultrasound was much appreciated for providing information that was seen as essential for adequate pregnancy management, such as the placental localization, assessing fetal growth, measuring umbilical cord blood flow and amount of amniotic fluid.

‘*Ultrasound is a tool that help us see how the pregnancy is progressing and gives us the answer, “Do we need to deliver* [the baby] *now, or should we continue monitoring the pregnancy?”’* (FGD 2)

Performing ultrasound examinations was described by the participants as very satisfying work that was of much value in pregnancy management especially for monitoring adverse pregnancy conditions.

*‘An ultrasound provides an overview of the conditions in the uterus (…), and repeating the* [ultrasound] *measurement, makes it possible to follow fetal development in a pregnancy where there are risks for complications’.* (FGD 3)

The midwives thought that in general pregnant women perceived the second trimester ultrasound examination as mandatory, and that very few pregnant women abstained from attending the examination. The participants had also noticed that an increasing number of women nowadays requested a first trimester ultrasound.

*‘The stream* [of requests] *for early ultrasound is absolutely huge. People want to look as soon as they are pregnant. That is not how it was before.’* (FGD 1)

Although the participants commented that some pregnant women seemed confident that their fetus was in good health as they felt fetal movements, others were seen to be worrying a lot about the fetus’ wellbeing, and wanted repeated ultrasound examinations for reassurance.

*‘Some are very aware of their body signs and can feel the fetus (...), and some want an ultrasound every fortnight in order to feel safe. (...) It appears to me that it has changed quite a lot.’* (FGD.1)

It was recognized that ultrasound examinations were performed not only for medical purposes, and that midwives sometimes performed an ultrasound examination to reassure pregnant women who were very worried about fetal wellbeing.

*‘We use it* [the ultrasound] *when there is a medical issue, and of course for the usual ultrasound screening, and sometimes for mothers who are very worried and in need of reassurance.’* (Interview 1)

When fetal deviations were detected at the ultrasound examination, midwives’ role to focus on the “normal” was thought to be a very valuable strategy for supporting expectant parents and help them to see all the other structures that were normal, not only the fetal deviations.

*‘It happens easily that the obstetricians only focus on deviations, and our* [the midwives’] *role can be to try to show the things that are normal and functioning in the rest of the body. And that may be the part that we midwives think a bit more about’.* (FGD 1)

*‘I remember that there was a pregnant woman who had one examination after another with the focus only on what was wrong, and thus she viewed the fetus as “a big defective kidney”. And the midwife did some lovely 3D images, and the fetus looked completely normal in these. It was really good for the couple. (…) In such situations our role can be to show what is normal’* (FGD 1)

The midwives described performing repeated ultrasound examinations in pregnancy as especially valuable for monitoring adverse pregnancy conditions.

*‘An ultrasound examination aims to provide an overview of the conditions in the uterus (…), and repeating the* [ultrasound] *measurement, makes it possible for you to follow the progress of a pregnancy and identify things that lead to complications.’* (FGD 3)

#### Places demands on midwives’ technical and counseling skills

It was pointed out that the medical value of ultrasound examinations was dependent on the operators’ knowledge and skills, and that the examination should only be performed by health professionals who had attended formal obstetric ultrasound courses. Repeated opportunities for ultrasound training to keep up with the development of the technique were also viewed as very important.

*‘It's important to have knowledge about what you can see* [with ultrasound]*, and to use the tool (…) so that you do the right things* [during an ultrasound examination]*. It can have consequences both ways, in terms of not detecting what is there and seeing things that aren’t really there* [false fetal deviations]*’.* (FGD 4)

Continuing technical developments in the field of obstetric ultrasound were perceived as both exciting and challenging, requiring repeated opportunities for training to keep up.

*‘I think there are great challenges. You learn something new constantly. There is always something that is going on* [in the ultrasound field]. *And the devices develop, our knowledge evolves, the ethics change and evolve constantly.’* (FGD 1)

Participants also expressed concerns that demands on the accuracy of the ultrasound diagnosis had increased, and that midwives could fear being blamed if they failed to detect deviations.

*‘You have it on your mind a bit, if there are things that you do not identify that you should have identified. (…) There is no acceptance of that nowadays, with the good [ultrasound] machines (…), so there may be the fear of not detecting deviations*’. (FGD 2)

It was acknowledged that health professionals performing ultrasound may become very preoccupied by looking at the ultrasound image, and there were concerns that midwives must be aware that expectant parents also need attention during the examination.

‘*You must not forget your professional* [role] *and look at the patients* [the pregnant women]*, and how they are really doing, the clinical part, because it’s easy to turn your back and sit with your face towards the ultrasound screen*’*.* (Interview 6)

It was stressed that information about severe malformation should be conveyed so that the expectant parents understood it was their choice to continue with the pregnancy or not, and that they did not feel pushed into a particular decision by the health professionals.

*‘You have to put it* [the information] *in such a way that they* [the expectant parents] *understand that they may choose. Because if you detect or identify a major malformation and the parents for ethical reasons certainly cannot imagine terminating the pregnancy, then they must be allowed to continue to full term’.* (FGD 3)

However, informing expectant parents when fetal deviations were detected was considered to be complex, as the midwives should not provide any diagnoses, but still need to communicate that the examination could not confirm that everything looked fine and that a physician has to be consulted.

*‘At the moment you see that there is something wrong, “How shall I communicate this?”’* (FGD 4)

There were concerns expressed about expectant parents’ expectations to be provided with a lovely image of their “child”, and their lack of understanding about the limitations of the ultrasound technique, which was perceived as quite common. These situations were said to place higher demands on midwives’ skills in performing accurate assessments. One example was the situation of overweight and obesity, as this can limit visualization of fetal autonomy and reduce the quality of the ultrasound image.

*‘When big women who come with exactly the same expectations of a good-quality examination* [as other pregnant women do] *and it is difficult to see because she is overweight, how do you inform her about this? I might not be able to do the examination as well as she expects, because of her being overweight*’ (FGD 1)

### Ultrasound contributes to the fetus being viewed as a ‘child’

This second category describes the midwives’ experiences regarding effects that ultrasound has had on the status of the fetus in pregnancy care and women’s obligations to the fetus during pregnancy. This category is described in the three following subcategories.

#### The ultrasound creates an image of a ‘child’

The development of ultrasound machines and fetal imaging was said to make the fetus appear like a child. The midwives commented that the more realistic the ultrasound image had become, the greater had become the perception of the fetus as a child, and they also felt that the fetus was now regarded as a person at an early gestational age.

*‘You dealt with the unknown, when very few* [pregnant women] *had an ultrasound. Today I notice it more, that I myself have some trouble seeing the fetus as a fetus, I realize I want to think of it as a child’.* (FGD 4)

It was admitted that health professionals were involved in this process of personification by providing expectant parents with copies of the ultrasound image, and placing ultrasound images of fetuses in public places.

*‘The media and the technological development have been a part of creating a different view of the fetus, and all these 3D and 4D* [ultrasound examinations]*, make them very baby-like. We too have a poster* [at the fetal medicine unit]*, that in a way personifies the fetus very much with its personality traits, you know.'* (FGD 4)

Midwives reported witnessing expectant parents starting to plan for things to buy for their “child” as soon as they saw the fetus on the ultrasound screen. This could also be considered very challenging especially when there were signs of fetal complications. One midwife described sometimes wanting to tone down expectant parents’ joy and conversation about plans for their “child”.

*‘If you see that there are twins and they are in the same sack, then you think “please help”* [how to convey that it is a high risk pregnancy]*. And then you say that there are twins, and then* [the expectant parents might say] *“Oh! What pram shall we buy?” And you think, “Will these twins be born* [alive]?”.’ (FGD 4)

It was suggested also that ultrasound could be used to enhance women’s bonding to the fetus in specific situations, such as when pregnant women used drugs or had psychiatric problems.

*‘I work with those in more difficult situations, such as drug abuse, or psychiatric and psychosocial issues. We use ultrasound as part of their interaction with the baby and it is a very good way to use ultrasound, to show the baby and to study a little how the parents behave when they see their baby in the uterus. And that's a very important part because it provides an early connection and interaction with the baby ’*. (FGD 5)

#### It makes you treat the fetus as a patient

The enhanced possibility to diagnose fetal conditions by ultrasound was reported to have entailed an increased focus on medical surveillance and interventions aimed to improve fetal health. It was further stated that fetuses were treated as patients needing a health assessment, and that midwives also had an important role to protect fetal wellbeing.

*‘Performing ultrasound is done to look for deviations and treat them, because we are both the fetus’ clinicians and advocates. It’s important therefore to find malformations to be able to prepare for taking care of this sick baby, and to do a good job and provide a good prognosis.’* (FGD 2)

It was felt that collaboration between obstetricians and pediatricians could enhance the results of medical management related to pregnancy complications. However, the decision-making was perceived as becoming more complicated as expectant parents and the different professional groups had become more involved.

*‘I feel that there are more* [professional] *groups now that are engaged in the decision making about when the fetus is considered viable. The mother is surely engaged in the decision, but at the same time there is the pediatrician and other professionals who provide guidance* (…), *and I think that is very difficult.*’ (FGD 1)

It was also said that there could be conflicting views between health professionals in obstetrics and pediatrics when the woman had a serious condition and that the obstetricians could suggest termination of pregnancy for the sake of the woman’s health, while the pediatrician focused on keeping the fetus in the uterus in order to enhance the prognosis for the fetus.

*‘I think that there may be some conflicts, because here, on the part of the fetal medicine specialists, one is accustomed to there being the choice to terminate a pregnancy with serious conditions, while the pediatricians, they think only of saving and treating, isn’t that right?’* (FGD 1)

Although maternal health was considered to be prioritized in obstetric care, midwives recognized that ultrasound enabled diagnosis of fetal conditions, which in turn could make health professionals postpone delivery for the sake of the fetus although this could mean that the woman’s health would be compromised.

*‘We are accustomed to putting the mother's health first and foremost but that is sort of a balancing act’* (FGD 5)

#### Only ‘irresponsible’ mums decline ultrasound examinations

The midwives believed that the ultrasound had opened up the opportunity for the fetus to be placed in a more central position within antenatal care, and that a strong focus on the fetus’ wellbeing could increase demands on pregnant women’s responsibilities in pregnancy. The increasing possibilities to diagnose and monitor fetal conditions by ultrasound were experienced as having entailed new demands on the pregnant woman to do what was best for the fetus. Although it was not common that women declined ultrasound or other examinations for fetal surveillance, it was mentioned that health care professionals could view pregnant women as irresponsible if they did not accept an offer of an ultrasound. Concerns were also expressed regarding potential risks that pregnant women’s autonomy would be restricted when obstetric ultrasound is a routine examination in maternity care.

*‘Now it's almost like if you do not accept the offer* (of an ultrasound examination)*, then you are a bad mother almost. (…) Are you irresponsible then? I don’t know.* (FGD 1)

Although it was agreed that pregnant women had the right to decide over their own bodies, the midwives admitted that pregnant women could be blamed if they did not accept recommendations of surveillance or medical interventions aimed at enhancing fetal health.

*‘We had one* [woman] *recently who did not want to take the medicine* [cortisone]*, and it was like "you must do it". At least most of us reacted like that when the mother didn't want to do it* [take the medicine] *even though it was best for the “child”.’* (FGD 5)

### Ambivalent feelings about obstetric ultrasound

This third category describes ambivalent feelings among the participants regarding obstetric ultrasound examinations which could lead to termination of pregnancy when fetal deviations were detected. While the ultrasound examination could be seen as a means for sorting out fetuses that are not perfect, the participants stressed that their intentions when performing ultrasound examinations were to provide support to expectant parents and optimize pregnancy outcome.

#### Ultrasound may be perceived as a selection method

Ambivalence was expressed among the midwives about the use of ultrasound to enable a decision to terminate a pregnancy at early gestations when the fetus did not have the favored gender or when fetal anomalies were detected. It was also recognized that the ultrasound examination could be perceived as a selection method that aims for perfection in human reproduction by rejecting imperfect fetuses.

*‘One cannot deselect because of gender. It would create problems in society. There are many ethical discussions surrounding ultrasound. There is a lot that can be revealed and which fetuses are we beginning to reject?’* (Interview.6)

There were also concerns expressed that the routine use of ultrasound and other tests for fetal anomalies could risk creating a society where only the perfect human was accepted, and that in the future people might feel that they do not have the option to refrain from terminating the pregnancy when fetal anomalies were detected.

*I do not think I would have wanted to have that choice* [pregnancy termination]! *But I think people might feel in the future that they don’t have any choices, and will just choose to reject what* [the fetal malformations]*, everybody else rejects. Will it be socially acceptable in a few years?* (FGD.4)

#### Ultrasound examinations are intended to optimize pregnancy outcomes

Midwives reported critical views in the community regarding midwives’ engagement in fetal anomalies screening, that midwives were thereby somehow implicated in rejecting fetuses that were not ‘perfect’. However, participants were at pains to point out that when performing an ultrasound their focus is always on optimizing pregnancy outcomes, not on the possibility of rejecting fetuses when anomalies are detected.

*‘Lots of people say, “So you work with some kind of sorting*” *No, I don’t do that! My focus is on what we can save, what we can help with. It’s great that we can. (…) We can do a blood transfusion instead of having a* [fetus with] *hydrops, and all the things you had to deal with before* (FGD.4)

Although the expectant parents’ choices regarding pregnancy management sometimes contradicted the midwives’ personal values, it was agreed among participants that expectant parents’ decisions should always be respected.

*‘You may feel that your own values are challenged, still you need somehow to find a way to help, for example when you find* [that the fetus has] *Down syndrome, and when they* [women] *then terminate it* [the pregnancy]*, so I feel that it goes a bit against my values but it’s not my role to say what is important and right for that couple.’* (FGD.2)

However the midwives also noted that performing ultrasound screening for fetal deviations may create conflicting feelings related to their own personal values, as detecting fetal anomalies could lead to termination of the pregnancy.

*'It’s a daily conflict with myself and the job. (…). We do perform a review of the organs, whether we like it or not. Then it's for checking that it looks alright, as far as we can see. Some might say that we're only looking for excluding defects.* (FGD.1)

## Discussion

It is well established that ultrasound plays an important role in the medical management of pregnancy [2], and that the examination is much valued both by expectant parents and antenatal care professionals [21], something which this study also confirms. An increasing demand for ultrasound examinations from expectant parents was also noted by the participants in this study. Performing obstetric ultrasound examinations were seen to place high demands on the health professionals’ technical and counseling skills. It was considered further that the advancements in ultrasound diagnosis had placed the fetus in a more central position in maternity care. Ethical concerns were raised regarding screening for fetal anomalies and the possibility of terminating the pregnancy when anomalies are detected, and that this could risk creating a society where only ‘perfect’ children are valued.

While ultrasound is highly valued both by expectant parents and healthcare professionals it has been suggested that there may be differences in their views and expectations of the ultrasound examination [22]. While healthcare professionals performing ultrasound aim to detect adverse fetal conditions and developmental deviations, expectant parents may focus on viewing “their unborn child” [22], and may perceive the ultrasound examination as a reassurance that everything is normal with the pregnancy [23].

Although no examination can guarantee that a fetus is healthy, an ultrasound examination may strengthen expectant parents’ confidence that the pregnancy will progress normally. It has been argued however that intense monitoring and registration of fetal status can promote the view of the fetus as vulnerable and in need of protection [24], and that this approach risks creating anxiety and guilt in pregnant women [24]. Providing ultrasound examinations for reassurance may also be of little value as the assessment is momentary and cannot ensure that the fetal situation is stable [25]. It has been noted however that Norwegian physicians may still view anxiety in women as a reason for performing ultrasound, although it is not listed as an indication for ultrasound in the national guidelines [15], a view that was also expressed by the midwives in our study. The Norwegian regulations for obstetric ultrasound screening and diagnoses have been criticized for being unclear and for thus allowing subjective assessments of indications for performing ultrasound examinations [15].

The importance of adequate knowledge and skills for healthcare professionals performing obstetric ultrasound was strongly emphasized in the FGDs and in the individual interviews. The quality of obstetric ultrasound has also been earlier described as very operator-dependent [26]. Post-graduate education in obstetric ultrasound for physicians and midwives has been shown to increase the standard of fetal anomaly screening [27], and basic theoretical and practical ultrasound courses are now obligatory in Norway and in the other Scandinavian countries [26]. It is suggested however that the ongoing development of ultrasound machines and of ultrasound technique may further increase demands on operators, and that advanced ultrasound training is needed for all healthcare professionals performing obstetric ultrasound, something which is not yet the case in all European countries [26]. There have also been calls for development of reliable measures for assessment of operators’ level of ultrasound competence and methods to ensure high quality ultrasound examinations [28]. Detection of fetal anomalies is known to create high levels of psychological distress in pregnant women [29], which places further demands on support for expectant parents in these situations. Although parental support needs to be individualized when adverse fetal conditions are detected, it has been shown that written and web-based information resources, and access to a reliable and informative website may also be of value [30]. The potential for these resources to support expectant parents as a complement to traditional counseling should be further explored.

This study shows that performing obstetric ultrasound can be very rewarding work for midwives and that they can experience ultrasound as helping expectant parents to connect with their “child” and gain reassurance about fetal health. However, screening for fetal anomalies may also conflict with some midwives’ ethical values regarding the possibility of pregnancy termination when fetal anomalies are detected. Concerns have been raised regarding an evolving lack of acceptance in society for having children with congenital anomalies [31]. While pregnancy ultrasound is much valued by healthcare professionals, critical views have been expressed regarding use of ultrasound in decisions about continuing a pregnancy or not [32, 33]. Ultrasound diagnoses of minor fetal deviations as soft-markers have been seen to be especially difficult to deal with, as these markers may create much anxiety in expectant parents although the findings in reality have little significance for fetal health [34, 35]. Advances in ultrasound imaging are further suggested to have given the fetus personal status and the position of a patient in pregnancy management [25]. Placing the fetus in a more central position in maternity care may however restrict pregnant women’s autonomy in situations when maternal and fetal needs conflict. The issue of balancing between maternal and fetal needs in pregnancy management was also raised in this study, where it was suggested that the midwife should act as an advocate for the fetus. The ultrasound examination, introduced in maternity care to assess maternal and fetal conditions of significance for pregnancy development, has now also become a means for expectant parents, not only to gain assurance about fetal well-being [36], but also to meet and connect with their future child [7, 37]. Performing ultrasound in pregnancy for non-medical reasons has been criticized however, for using medical technology to provide enjoyment for the expectant parents, i.e. “entertainment ultrasound” [38]. It has been suggested though that this may be beneficial for social reasons in specific situations such as when the fetus is found to have a lethal condition [39].

### Trustworthiness

To strengthen credibility in this study the participants were recruited from five different hospital-based obstetric clinics at various levels of the health care system. They differed in characteristics such as age and various lengths of experience in performing obstetric ultrasound. All participants were female midwives, which was expected considering that there are very few males in this healthcare profession in Norway. Further, dependability was ensured by using an interview topic guide, which meant that the same topics were brought up for discussion in all FGDs and interviews. Using data from both FGDs and individual interviews may provide divergent results, however there were no topics brought up in the individual interviews by the two participants that had not been raised in the FGDs. Data collection was performed by two Swedish researchers, which could risk some misinterpretation of the Norwegian language. However the two interviewers (AÅ and SH) were both familiar with Norwegian, which is closely related to Swedish, and two of the authors (TF and ED) working in the Norwegian health system. After each FGD and interview the findings were discussed between researchers (AÅ, IM and SH). A detailed description of the study context, setting, participants, data collection and analysis is included to improve transferability. Though we are aware that our findings may not be widely generalizable, the results are certainly relevant to the context of maternity care services in Norway due to participant recruitment from a variety of hospital settings. It is also likely that they have some applicability for maternity care professionals in other western countries. Another limitation is that perspectives of pregnant women and their partners were not included in this study. Even though this was not within the scope of the study, their views would certainly provide additional perspectives on the use of ultrasound in pregnancy management.

## Conclusions

This study shows that performing obstetric ultrasound can be rewarding for midwives, who perceive the examination as a means to assist expectant parents and optimize pregnancy outcomes. Health professionals’ knowledge and technical skills are seen by the midwives as crucial for ultrasound examinations to be valuable. The midwives raised ethical concerns regarding the possibility that pregnancies with “imperfect” fetuses may be terminated as a result of the ultrasound examination. The midwives also noted that expectant parents may place much focus on the social aspects of the examination, seeing and connecting with their expected child, something which seemed to challenge the midwives’ professional and clinical role. More research needs to focus on the information given to prepare prospective parents in order to better understand the role and the potential outcomes of the ultrasound examination. It seems impossible however to avoid the social aspects of obstetric ultrasound i.e. expectant parents’ meeting and connecting with their “baby” during the examination. Therefore, routines need to be developed so that the medical information and counseling provided to expectant parents during the ultrasound examination, also acknowledge expectant parents’ desire for communication concerning their impressions and feelings when viewing the ultrasound imaging of their fetus.

## Additional files

Additional file 1: Key domains in the CROCUS interview guide (DOCX 17 kb)

Additional file 2: COREQ (COnsolidated criteria for REporting Qualitative research) Checklist (DOCX 23 kb)

## Abbreviations

CROCUS: Cross Country Ultrasound Study
