# Supplementary material for: A much valued tool that also brings ethical dilemmas - a qualitative study of Norwegian midwives’ experiences and views on the role of obstetric ultrasound
Source: BMC Pregnancy Childbirth. 2019 Jan 16;19:33. doi: 10.1186/s12884-019-2178-x (PMC6335783; doi:10.1186/s12884-019-2178-x)
Supplement: Supplementary file 1 — Key domains in the CROCUS interview guide (DOCX 17 kb) [file 12884_2019_2178_MOESM1_ESM.docx]

**Supplementary File 1. Key domains in the CROCUS interview guide**

| **Key domains** | The midwives’ experiences and views of:   - The role of obstetric ultrasound for clinical management of complicated pregnancy. - The role of obstetric ultrasound in comparison to other surveillance methods during complicated pregnancy. - Clinical situations where the interests of maternal and fetal health conflict. - Whether the woman may be considered to act as an instrument for fetal treatment. - If/when the fetus can be regarded as a person. - Situations where the fetus has been regarded as a patient with his/her own interests. - Their professional role in relation to other occupational groups working with obstetric ultrasound examinations or the outcomes of these examinations. - Their perception of the community’s views of obstetric ultrasound. - Their thoughts about the future in relation to the theme ‘Genetic engineering’. - Other issues in relation to ethical aspects of the use of obstetric ultrasound. |
| --- | --- |
